# Exploring the emerging role of the microbiome in cancer immunotherapy

**DOI:** 10.1186/s40425-019-0574-4

**Published:** 2019-04-17

**Authors:** Jessica Fessler, Vyara Matson, Thomas F. Gajewski

**Affiliations:** 1 Department of Pathology, The University of Chicago, Chicago, IL USA; 2 Department of Medicine, Section of Hematology/Oncology, The University of Chicago, 5841 S. Maryland Ave., MC2115, Chicago, IL 60637 USA

**Keywords:** Immunotherapy, Gut microbiome, Anti-PD-1, Immune checkpoint blockade, 16S rRNA gene sequencing, Metagenomics, Germ-free mice, Microbiome-based therapy

## Abstract

The activity of the commensal microbiota significantly impacts human health and has been linked to the development of many diseases, including cancer. Gnotobiotic animal models have shown that the microbiota has many effects on host physiology, including on the development and regulation of immune responses. More recently, evidence has indicated that the microbiota can more specifically influence the outcome of cancer immunotherapy. Therapeutic interventions to optimize microbiota composition to improve immunotherapy outcomes have shown promise in mouse studies. Ongoing endeavors are translating these pre-clinical findings to early stage clinical testing. In this review we summarize 1) basic methodologies and considerations for studies of host-microbiota interactions; 2) experimental evidence towards a causal link between gut microbiota composition and immunotherapeutic efficacy; 3) possible mechanisms governing the microbiota-mediated impact on immunotherapy efficacy. Moving forward, there is need for a deeper understanding of the underlying biological mechanisms that link specific bacterial strains to host immunity. Integrating microbiome effects with other tumor and host factors regulating immunotherapy responsiveness versus resistance could facilitate optimization of therapeutic outcomes.

## Background

The human body is a complex ecosystem inhabited and influenced by an abundance of microorganisms including bacteria, yeast, fungi, protozoa, archaea, and viruses, all of which collectively constitute the commensal *microbiota*. The commensal microbiota and the human host have co-evolved in a mutualistic relationship, in which each benefits the fitness of the other and the two can be collectively viewed as a superorganism. Much recent research has focused on the bacterial component of the microbiota. On average, a healthy human body is comprised of approximately 30 trillion cells and is inhabited by approximately 39 trillion bacterial cells [1]. The collection of genes within the commensal microbiota is defined as the commensal *microbiome* and vastly outnumbers human genes. The microbiota is capable of synthesizing or transforming a wide variety of metabolites, including hormones, essential vitamins, and other bioactive compounds, which cannot be otherwise acquired by the host [2]. These metabolites can modulate various biological functions, most notably the immune and nervous systems [3]. Alterations in the normal microbiota have been reported to contribute to the development of many diseases [4–15]. In the cancer context, some specific bacteria have been demonstrated to be involved in the process of carcinogenesis [15]. In addition, the microbiota has also been implicated in modulating the efficacy and toxicity of cancer therapy, including chemotherapy and immunotherapy [16]. Preclinical data suggest that modulation of the microbiota could become a novel strategy for improving the efficacy of immune-based therapies for cancer, in particular checkpoint blockade approaches targeting the CTLA-4 and PD-1 pathways [17, 18].

### Establishment of commensal bacterial colonization in the human host

In adults the microbiota consists of about a dozen phyla, primarily Firmicutes and Bacteroidetes, followed by Actinobacteria, Proteobacteria, Fusobacteria, and others [19]. The relative proportions of these phyla vary between individuals and between anatomical sites. The GI tract is considered the most impactful site of host-microbe interactions. Various factors can influence the composition of the gut microbiota in a given individual, such as the composition of the maternal microbiota, mode of infant delivery (vaginal vs. C-section), diet, exposure to antibiotics and other medications, germline genetics of the host, and environmental factors [20]. Initial microbial exposure may occur as early as in utero, where the GI tract of the fetus may first be colonized by maternal bacteria through ingestion of amniotic fluid [21, 22]. After vaginal delivery, the neonatal microbiota resembles the mother’s vaginal microbiota and is undifferentiated across anatomical sites [23], but subsequently becomes shaped by the selective pressure of site-specific factors and by 3 years of age, an adult-like intestinal microbiota dominated by Firmicutes or Bacteroidetes is established. After this age, the microbiome composition in a healthy individual reaches a relatively stable state with minor fluctuations in physiological conditions, but strong and prolonged perturbations can occur in disease conditions or with antibiotics. At the species level there is enormous inter-individual heterogeneity in gut microbiomes, which has hindered efforts for clearly defining a core microbiome shared between healthy individuals. It has been suggested that the functional capacity of the microbiota, as depicted by abundances of genes involved in metabolic pathways, could constitute a metric better suited to define a core healthy microbiota [19, 24]. Indeed, the basic categories of metabolic pathways were more evenly represented across individuals as compared to bacterial taxonomy [19]. It remains to be determined whether this shared set of metabolic pathways is the major characteristic of a healthy microbiota.

### Next generation sequencing methods in microbiome studies

Culturing of bacterial strains has been central to classical microbiology and has enabled the study of individual pathogens and some commensal bacteria. For most commensal bacteria, culture methods had not been optimized for their in vitro isolation and study. With recent improvements in methodology, a large proportion of commensal bacteria is now considered culturable [25, 26]. *Culturomics* is a strategy which incorporates multiple culture conditions, coupled with MALDI-TOF mass spectrometry and/or 16S ribosomal RNA (rRNA) or total genome sequencing for bacterial identification [27, 28]. This high-throughput approach can enable isolation and identification of commensals for further manipulation and mechanistic studies.

The most common method for taxonomic characterization of complex bacterial communities is based on selective amplification and sequencing of part of the gene encoding the 16S rRNA, part of the small ribosomal subunit in prokaryotes. This is a ubiquitous 1.5 kb gene, containing conserved sequences and hypervariable regions (nine regions: V1-V9), the latter being useful for bacterial taxonomic classification, as originally described by Woese and colleagues [29]. In the first step of this technique, a pair of universal primers targeting conserved sequences flanking a hypervariable region are used to generate an amplicon library, which is then sequenced. To account for sequencing errors, amplicons which share sequence similarity above a certain threshold are grouped into operational taxonomic units (OTUs). A representative amplicon is selected from each OTU bin and assigned a taxonomic identity based on cross-referencing to pre-existing databases [30–32]. All other amplicons in the OTU are also assigned the same identity. Thus, OTU binning can artificially decrease the observed diversity of a microbial community [33] and alternative methods for analysis have been proposed [34–36]. Because bacterial identification is based on a portion of the 16S rRNA gene, species level resolution is usually not feasible with this method and identification is typically limited to family or genus level [37]. Another consideration in 16S analyses is that most bacteria contain multiple copies of the 16S rRNA gene, which can lead to inaccurate quantitation of bacterial cells [38]. Additional bias can be introduced in the amplification step, depending on the choice of primers. Despite these limitations, the low cost and high-throughput potential of this technique make it the most commonly used for initial descriptive data.

Metagenomic shotgun sequencing generates short reads representing the whole genomic content within an environmental sample and is considered less biased than 16S rRNA gene amplicon sequencing, because it does not contain a PCR amplification step. However, this can result in contamination with human genomic DNA and requires higher sequence coverage to detect bacterial species of low abundance. This necessitates additional data storage, computing power, and more sophisticated analysis pipelines. Errors can also be introduced in the downstream analysis at the step of genome assembly or gene prediction [39]. Various bioinformatic tools have been developed for metagenome assembly, and databases have been established for gene prediction, but there is no consensus on the best strategy [40]. Compared to 16S rRNA gene amplicon sequencing, superior resolution down to species and strain level identity is feasible with shotgun sequencing because multiple marker gene sequences are used for taxonomic annotation [37]. This approach can also be used to characterize non-bacterial compartments of the commensal microbiota, including archaea, fungi or viruses. Another advantage of shotgun sequencing is that it can be used for characterization of the functional capacity encoded by the microbiome using gene prediction tools and databases [40]. By contrast, functional capacity can only be inferred indirectly from 16S rRNA amplicon sequencing data [41–43]. Each of these sequencing methods has its limitations, but the two can be integrated to improve the accuracy of bacterial identification and quantitation [44].

### Impact of the commensal microbiota on immunity: insights from gnotobiotic mouse models

The role of the commensal microbiota in modulating host physiology becomes particularly evident when conventionally raised specific pathogen-free (SPF) mice are compared to germ-free (GF, axenic) mice. GF mice are defined as devoid of detectable microbiota during their life. The term *gnotobiotic* pertains to animals with known (defined) microbiota composition and encompasses GF, as well as ex-GF animals colonized with defined microbial communities. The commensal microbiota broadly impacts host physiology, and this has been mainly shown in studies with GF mice, which have inefficient energy extraction from the diet, abnormal fluid balance and electrolyte status, and disturbances in liver, lung, cardiovascular system, endocrine organ, nervous system, and immune system functions [45, 46].

#### Impact on local immunity

The gut microbiota is intimately involved in the development and regulation of the immune system, especially with respect to local mucosal immunity. This has been demonstrated in GF mice, which show deficiencies in the gastrointestinal immune compartment rendering them more susceptible to infections. However, such deficiencies can be corrected by colonization with commensal bacteria. For instance, in GF mice, the mucus-producing goblet cells are fewer and smaller. As a result, the mucus layer, the first line of defense against pathogens in the intestine, is thinner and has a different mucin composition [47, 48]. Additional examples of GI immune defects in GF mice include: 1) smaller mesenteric lymph nodes (MLN) and abnormal high endothelial venules with poor lymphocyte binding [49]; 2) fewer and smaller Peyer’s patches which lack germinal centers [50, 51]; and 3) lack of lymphoid follicles in the intestinal lamina propria (LP), but presence of nascent cryptopatches which can develop into functional isolated lymphoid follicles upon microbial colonization [52–54]. These local immune deficiencies are accompanied by a decreased number of LP CD4^+^ T cells, plasma cells, and decreased IgA production leading to further impaired intestinal barrier function [55, 56]. The presence of commensal bacteria is required not only for normalizing the LP CD4^+^ T cell numbers, but also for proper programming of the local Treg/Th17 balance. GF mice are almost completely devoid of Th17 cells, but have increased frequency of FoxP3^+^ T cells [57].

#### Impact on systemic immunity

Systemic innate immune modulation is also influenced by the commensal microbiota, with multiple lines of evidence indicating stimulatory effects on myelopoiesis at the level of granulocyte-macrophage progenitors in the bone marrow and in the periphery, as well as on the function of DCs, macrophages, and neutrophils (reviewed in [58]). In many cases, these systemic effects have been attributed to circulating bacteria-derived molecules (microbe- or pathogen-associated molecular patterns, MAMPs and PAMPs, respectively), such as lipopolysaccharide (LPS), peptidoglycan, or flagellin, which when recognized by pattern-recognition receptors (PRRs) on innate immune cells, can signal via a MyD88-dependent pathway to enhance systemic innate immune cell responsiveness [58]. Bacterial metabolites, such as short-chain fatty acids (SCFA), the products of dietary fiber fermentation by the microbiota, have been implicated in stimulating DC generation in the bone marrow and their phagocytic capacity [59]. Systemic adaptive immunity is also stimulated by the presence of commensal bacteria, particularly the proper development of distant (non-mucosal) lymphoid tissues, such as the spleen and peripheral lymph nodes. This is evidenced by the poorly developed B cell follicles and T cells zones in these organs in GF mice, leading to decreased IgG levels in the serum [60, 61]. Commensal bacteria are also required for proper programming of the Th1/Th2 balance and in GF mice there is a bias towards Th2-type allergic responses, which can be corrected by colonization with commensal bacteria [62].

#### Specificity of microbiota-mediated immune programming

Different members of the commensal microbiota are not equivalent in their capacity to polarize T cell responses. For instance, in SPF mice the group of segmented filamentous bacteria (SFB), which colonize the mouse terminal ileum and adhere to the epithelial cells, are particularly potent inducers of Th17 cell differentiation [63]. SFB are not found within the human microbiota, but further studies have shown that other bacteria derived from human fecal samples are also capable of adhering to the epithelial layer and inducing Th17 cells when transferred to mice [64–66]. By contrast, Treg differentiation and function are strongly induced by *Bacteroides fragilis* [67] and *Clostridium* clusters XIVa, IV, and XVIII [68, 69]. Polysaccharide A (PSA) from the capsule of *B. fragilis* can polarize towards Th1-type responses [62]. Higher Bacteroidetes/Firmicutes ratio resulting from high-fiber diet increased the levels of circulating SCFAs and alleviated Th2 cell-mediated allergic airway inflammation by reducing the capacity of lung-resident DCs to drive Th2-type responses [59]. Monocolonization of GF mice with 52 different human commensal bacteria demonstrated that most of the species were capable of inducing alterations in the frequency and function of immune subsets within the intestinal LP, Peyer’s patches, MLN, and spleen. Some more notable effects were alterations in cytokine production in the LP and in frequencies of Treg, pDC, CD103^+^ dendritic cells (DCs), macrophages and mononuclear phagocytes [66]. Notably, many species were able to translocate to the MLN and spleen [66]. This is likely an artifact of the model, due to the poor intestinal barrier function in GF mice. Therefore, the mechanisms leading to the observed alterations in immune cell subset composition, especially those seen systemically, may not in all cases reflect the physiological state.

#### Practical considerations in the use of germ-free mouse models

SPF mice have been used to gain valuable insight in the impact of microbiota-host interactions on host physiology in health and disease. When it comes to clinical translatability, a question that arises regarding the degree of similarity between the microbiomes of humans and laboratory mice. Although a direct comparison between datasets from different studies can be blurred by differences in analysis platforms and protocols, a general consensus exists that on a phylum through family level, the microbiomes of SPF mice and humans are similar with both species being predominantly colonized by Bacteroidetes and Firmicutes [70, 71]. Comparison between datasets on a deeper taxonomic level is challenging because of limited representation of microbial genes in the current databases causing difficulties with genus, species and strain level annotation. A study comparing microbial metagenomes of humans and SPF mice of different genetic backgrounds and housed in different facilities showed that only 4% of microbial gene sequences were shared between humans and mice. Despite that discordance, functional annotation of the mouse and human microbiomes using the KEGG database revealed that 85% of the annotated gene orthologs were shared between mouse and human microbiomes [72]. Therefore, the murine organism as a host appears to have similar functional requirements for the commensal microbiota, which makes it an appropriate recipient of human microbiota for studying its effects on host physiology. A high value of GF mice in microbiome research is their utility in generating purely human microbiota-associated mouse models for studying microbe-host interactions and demonstrating causal effects of the microbiota on the health/disease states of the host. Indeed, successful transfer of microbiota from humans to GF mice often imprints the human health phenotype onto the murine recipient.

There are some differences between mice and humans which might affect the efficiency of human gut microbiota engraftment into mice or their spatial establishment throughout the GI tract. A potentially relevant difference in GI tract anatomy is the presence of a non-glandular fore-stomach in mice, which takes up two thirds of the stomach, has no secretory activity, and serves for temporary food storage. This allows for food to be ingested in bulk, but to be released for downstream digestion more gradually according to energy demands. The lack of gastric secretions in the fore-stomach results in higher pH of its contents (pH 4.8) [73] and the overall pH in the mouse stomach is 2.7–4.1, while in humans it can be as low as pH 1 [71]. The milder pH and the abundance of oligosaccharides in the mouse fore-stomach provide conditions for the bloom of *Lactobacillae*, whereas in humans, the stomach contains mainly *Streptococcus*, *Prevotella spp*. and *Helicobacter pylori* [71, 73]. Another difference is the presence of circular folds (*plicae circularis*) in the human small intestinal mucosa, which are absent in mice [71, 74]. These structures could provide additional niche for mucus-associated bacteria [71]. Mice also have a relatively large cecum, where microbial fermentation of indigestible fiber takes place, while in humans the cecum is small and of uncertain importance [74]. In humans, fermentation and production of vitamins K and B and SCFA occur in the colon, which is segmented into pouches (haustra). The cecal appendix in humans is enriched in gut-associated lymphoid tissue and in microbial burden and has been hypothesized to serve as a reservoir of beneficial bacteria which may replenish the microbiota after diarrhea or other disturbances [75]. In mice, the appendix does not exist as a separated structure. Additional differences in GI tracts of humans and mice that might affect the fidelity of human microbiota transfer to mice include overall lower pH and oxygen tension in the mouse intestine, as well as differences in the glycan profile of the mucus, which might affect the growth of mucus-utilizing bacteria. Apart from differences in GI tract, the inability of some bacterial species to survive the conditions of the transfer, including storage outside the host, oxygen exposure, and longer time spent in the stomach, may also limit the fidelity of reconstitution in mice. Differences in diet between human donor and mouse recipient could additionally result in skewed engraftment profiles. The sex of the recipient mouse has also been shown to affect colonization fidelity [76].

GF mice have many physiological defects, which can become a confounding factor in microbiome studies. Notably, due to compromised intestinal barrier function and immature immune system in GF mice microbial colonization could result in systemic translocation and abnormal magnitudes and sites of microbe-host interactions [66]. A more physiologically relevant mouse colonization would be the acquisition of experimental microbiota from the mother at birth. Thus, the offspring from artificially colonized by gavage ex-GF mice, can be used for experimentation. It has been shown that the microbiota from artificially colonized ex-GF mice bred in an isolator can be vertically transmitted to generations F1 and F2 without significant drift between generations [77]. The use of such offspring mice could also capture effects of microbiota-mediated epigenetic immune programming occurring in utero. In addition to proper guiding of immune system maturation, such natural colonization of offspring mice with a functionally complex microbiota could eliminate other confounding factors such as the metabolic and endocrine abnormalities characteristic of GF mice. Therefore, an important experimental tool is to generate gnotobiotic mouse colonies maintaining a stable and defined microbiota derived from individual human subjects, functionally recapitulating the complex SPF microbiota and normalizing mouse physiology [78]. Towards this goal, it has been shown that a small number of culturable bacterial strains can cover most of the functional potential of the gut microbiome [79, 80]. Individual strains of interest can then be introduced and their immunomodulatory roles can be studied in the context of more physiologically relevant conditions [80].

An alternative to using GF mice as human microbiota recipient is the use of antibiotic-treated SPF mice. Although SPF mice with intact microbiota are generally not receptive to human microbiota, engraftment can be substantially improved with certain antibiotic regimens, which deplete the bulk of the pre-existing commensals, thus opening up an niche for subsequent colonization [81, 82]. Such models can be a useful alternative in mechanistic studies with some mouse strains of genetically engineered mouse models unavailable in GF status. However, the potential contribution of residual non-depleted mouse microbiota should be considered in such experimental settings, including its influence not only on the host but also on the acquired human microbes.

When interpreting results from experiments with GF mice, it should also be considered that even though GF mice are devoid of detectable viable microbiota, they are exposed to microbial residues (MAMPs, PAMPs, or antigens) derived from dead bacteria in sterile diet and bedding [83]. If present in sufficient quantities, these molecules could theoretically affect immune functions in a similar manner as do intact viable bacteria. For instance, MAMPs/PAMPs can be recognized by PRRs on intestinal epithelium or mucosal immune cell subsets leading to downstream signaling. Bacterial antigens can be sampled directly from the intestinal lumen by DCs or can be transported to LP antigen-presenting cells (APCs) via passage through goblet cells. APCs, in turn, can migrate to MLN and activate adaptive immunity. Bacterial antigens may also be taken up by M cells to stimulate plasma cell development and IgA secretion in Peyer’s patches. Because GF mice have poor barrier function, MAMPs/PAMPs and antigens might also translocate into the circulation and affect systemic immunity. Commonly used sterile diets can have a range of levels of microbial residues. For instance, LPS content, as a measure of overall bacterial contamination in diets, shows a range of 1–100 EU/μg [84]. A sterile diet rich in microbial residue can induce maturation of the immune system in a similar manner (albeit less prominently), as does colonization with commensal bacteria, with particularly strong impact on CD4^+^ T cells and Treg cells in the MLN and IL-4 and IL-12 cytokine responses in spleen cells [84]. Indeed, a sterile chow that contained high level of microbial residues resulted in decreased Th2-type response to allergic sensitization of GF mice compared to a sterile diet that was poor in microbial residues [83]. Use of chemically defined ultra-filtered diet, rather than conventional sterile chow could uncouple the effects of microbial colonization from those of dietary microbial residue exposure.

## Evidence linking the gut microbiome to cancer immunotherapy

Multiple studies support that gut microbes can profoundly influence the potency of immunotherapy and some chemotherapies with immunostimulatory functions (summarized in Table 1). Pioneering work in this field found that intestinal microbiota was essential for optimal responses to CpG-oligonucleotide immunotherapy which activates innate immune cells through TLR9 [85]. Similarly, the gut microbiota was found to shape the anti-cancer immune response by stimulating generation of a specific subset of “pathogenic” Th17 (pTh17) cells and memory Th1 immune response after treatment with immune-stimulatory chemotherapy cyclophosphamide [86]. Certain bacterial taxa in patients with hematologic malignancies are associated with efficacy of allogeneic hematopoietic stem cell transplantation (allo-HSCT) and decreased risk for graft-versus-host disease (GVHD) following therapy [87, 88]. Initial evidence for the contribution of specific microbes to immune checkpoint blockade (ICB) immunotherapy, including CTLA-4 and PD-1/PD-L1 blockade, was demonstrated in mouse models [17, 18]. *B. fragilis* was reported to enhance anti-CTLA-4 efficacy via a proposed mechanism involving the activation of Th1 cells with cross-reactivity to bacterial antigens and tumor neoantigens [18]. Oral administration of *Bifidobacterium* increased tumor infiltration and IFN-γ production by CD8^+^ tumor-specific T cells and improved both basal tumor control and anti-PD-L1 efficacy via a proposed mechanism involving increased activation of splenic and intratumoral DCs [17]. These mouse studies established the importance of the microbiome in cancer ICB therapy and inspired clinical pursuits to assess the microbiome’s impact on anti-CTLA-4 and anti-PD-1/PD-L1-based therapies in patients.

Results from multiple institutions have contributed to the growing consensus that the gut microbiome is linked to immunotherapy efficacy in cancer patients [44, 89–92]. DNA sequencing of stool samples collected prior to checkpoint blockade therapy identified an association between gut microbiome composition and subsequent therapeutic response. Distinct bacterial taxa were overrepresented in responder (R) patients, whereas other bacterial sequences were over-represented in non-responder (NR) patients. Importantly, only some of these identified bacteria were consistent across multiple studies. This discrepancy may reflect discordant biology—the patient populations were from geographically distinct locations, with potentially dissimilar environmental and genetic factors—but also may be explained by technical differences, such as fecal collection, storage and DNA extraction and sequencing methods, as well as downstream bioinformatic analysis. Moving beyond correlative studies, human microbiota “avatars” (GF mice colonized with patient stool-derived commensals) have been used to show the mechanistic contribution of the microbiota to treatment response. Mirroring patient data, mice reconstituted with R patient fecal material showed greater benefit from checkpoint blockade than mice colonized with NR fecal samples [44, 89, 90]. Beyond clinical efficacy rate, immune-related toxicity of ICB has also been linked to the composition of the gut microbiome. Based on stool samples collected from patients treated with an anti-CTLA-4 antibody, bacteria in the Bacteroidetes phylum were associated with lower incidence of treatment-induced colitis [93].

**Table 1:** Studies linking the gut microbiome composition to efficacy of cancer therapy. The table summarizes major findings from clinical and preclinical studies pointing to a link between gut bacteria and therapeutic outcomes in the context of various cancers and therapeutic regimens

Major finding	Mouse or Human data	Cancer/Therapy	Reference
Chemotherapy with immunostimulatory properties			
*Akkermansia muciniphila* abundance in baseline stool samples was associated with response to ICB	Mouse	Various cancer models/Cyclophosphamide immunostimulatory chemotherapy	[86]
Presence of intratumoral gammaproteobacteria was associated with resistance to gemcitabine chemotherapy	Human; Mouse	Pancreatic ductal adenocarcinoma/ Gemcitabine immunostimulatory chemotherapy	[94]
Immunotherapy			
Commensal microbiota was required for optimal response to therapy	Mouse	Various cancer models/ CpG-oligonucleotide + anti-IL-10R antibody and platinum chemotherapy (oxaliplatin)	[85]
Total body irradiation disrupted intestinal barrier and improved outcome of T-cell based therapy by a mechanism dependent on LPS/microbe translocation and TLR4 signaling	Mouse	Melanoma/Adoptive T cell transfer	[97]
*Eubacterium limosum* abundance was associated with decreased risk of relapse or disease progression	Human	Hematologic cancers/Allo-HSCT	[88]
*Blautia* abundance was associated with increased overall survival and reduced risk of GVHD	Human	Hematologic cancers/Allo-HSCT	[87]
*Bacteroides* abundance was associated with resistance to ICB-induced colitis	Human	Metastatic melanoma/Anti-CTLA-4	[93]
*Bacteroides* abundance was associated with response to ICB	Mouse; Human	Metastatic melanoma/Anti-CTLA-4	[18]
*Bifidobacterium* abundance was associated with improved spontaneous anti-tumor immunity and response to ICB	Mouse	Melanoma/Anti-PD-L1	[17]
*Faecalibacterium* and other Firmicutes abundance in baseline stool samples was associated with response to ICB; Bacteroides abundance was associated with poor responsiveness to ICB	Human	Metastatic melanoma/Anti-CTLA-4	[92]
*Bacteroides caccae*, *Faecalibacterium prausnitzii*, *Bacteroides thetaiotaomicron*, *Holdemania filiformis*, and *Dorea formicogenerans* were associated with response to ICB	Human	Metastatic melanoma/Anti-PD-1; Anti-CTLA-4	[91]
*A. muciniphila* abundance in baseline stool samples was associated with response to ICB	Human; Mouse	Non-small cell lung cancer; Renal cell carcinoma/Anti-PD-1	[89]
Higher microbiome richness, Clostridiales, Ruminococcaceae, and Faecalibacterium abundance, and enrichment in genes involved in anabolic pathways in baseline stool samples were associated with responsiveness to ICB	Human; Mouse	Metastatic melanoma/Anti-PD-1	[90]
Several dozen bacterial species in baseline stool samples were differentially enriched between patients with strong vs. poor responsiveness to ICB	Human; Mouse	Metastatic melanoma/Anti-PD-1	[44]

## Deciphering the biological mechanism of microbiome mediated immune modulation

These findings linking the gut microbiome to immunotherapy efficacy only scratch the surface of this complex interaction. Determining the biological mechanisms is critical for moving towards therapeutic manipulation of the microbiota to optimize patient response. Tractable mouse models are being utilized to explore the causal role gut bacteria play in treatment efficacy.

**Fig. 1:**
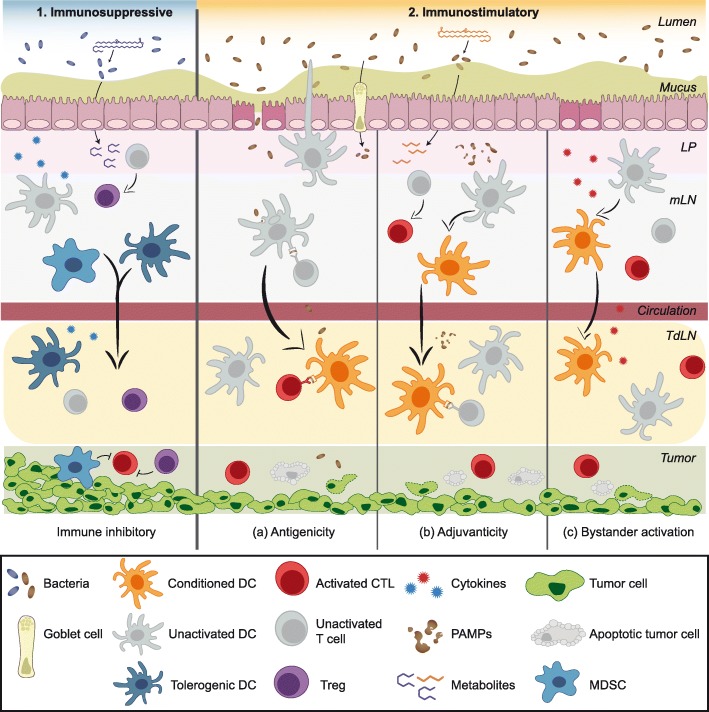
Possible mechanisms linking the gut microbiota to anti-tumor immunity. The composition of the gut microbiome may impact immunotherapy efficacy by either acting as (1) an immunosuppressive or (2) an immunostimulatory factor via various non-mutually exclusive mechanisms. (1) Certain commensal bacteria may suppress anti-tumor immunity by skewing immune subset balances towards suppressive phenotypes such as Tregs and MDSCs. Locally in mucosal sites, induction of immunosuppressive cells could be mediated by cytokines released by host cells (such as gut epithelium or immune cells) in response to microbial sensing. Immunosuppressive effects in distant sites, such as active immunosuppression in the TME, could be mediated by trafficking of locally induced suppressor cells. Additionally, bacterial metabolites with immunosuppressive properties might be released into the circulation and promote immunosuppressive cell functions in the TdLN and TME. Chronic inflammation caused by continuous stimulation by PAMPs/MAMPs or epithelial injury could also ultimately contribute to immunosuppression over time. (2) The immunostimulatory effects of the gut microbiota could be mediated by augmented antigenicity, adjuvanticity, or bystander T cell activation. (a) Antigenicity: Cross-reactive T cells driven by bacterial antigens that additionally recognize tumor-associated antigens is one conceivable mechanism. Luminal bacteria or bacterial antigens can be internalized by DCs in the LP via trans-endothelial dendrites extending through the epithelium into the lumen. Goblet cells and M cells can also serve as portals to deliver bacterial antigens to mucosal APCs. Alternatively, disruption of barrier function may allow for the translocation of luminal bacteria and bacterial antigens. Antigen-loaded DCs can migrate from the LP to the MLN and possibly to distant sites such as the TdLN, where they may prime cross-reactive anti-tumor CD8^+^ or CD4^+^ T cells, enhancing cytotoxic T lymphocyte (CTL) function in the TME. (b) Adjuvanticity: PAMPs/MAMPs may condition DCs to be more potent T cell activators, for instance by upregulating costimulatory molecule expression, enhancing antigen presentation, or boosting type I IFN production. Some microbial metabolites could alter immune cell function epigenetically or otherwise to poise innate and adaptive cells in a heightened activation state. (c) Bystander activation: A heightened inflammatory state in the TME driven by pro-inflammatory cytokines released in response to bacterial stimuli may contribute to tumor cell killing by T cell help provided by bacteria-specific T cells to tumor antigen-specific T cells

When it comes to exploring the possible mechanisms of microbiota-mediated modulation of anti-tumor immunity, two general questions arise. First, what is the nature of the *messenger*, which delivers a signal from the GI tract to the tumor and/or tumor-draining lymph node (TdLN)? Such a messenger would be able to enter the circulation in order to access the distant tumor site and can be classified as microbiota- or host-derived cell (live microbes or host immune cells) or molecule (MAMP/PAMP, microbial metabolite, or host cytokine). The second question is what is the nature of the *immune effect* that the messenger confers within the tumor? An immunosuppressive effect could be achieved by augmenting regulatory functions (Tregs, MDSCs, tumor-associated macrophages) or directly inhibiting anti-tumor immunity; an immunostimulatory effect could be achieved by alleviating regulatory functions or stimulating anti-tumor T cell responses (via antigenicity, adjuvanticity or bystander activation). The exact mechanisms of microbiota-mediated effects on tumor growth and efficacy of immunotherapy are only beginning to be understood. Figure 1 summarizes these hypothetical scenarios and early evidence is discussed below.

### Live bacteria or MAMPs/PAMPs as messengers

Commensal bacteria have been identified in extra-gastrointestinal tissues typically considered to be sterile. Notably, Geller et al. identified bacteria within the TME in human pancreatic ductal adenocarcinoma [94]. In this study, viable bacteria were hypothesized to gain access to the cancerous lesions via a retrograde migration from the duodenum towards the pancreatic duct and were shown to decrease gemcitabine chemotherapy efficacy by metabolizing the active form of the drug. In terms of impact on immune function, it was experimentally shown that bacterial translocation into the MLN and spleen generated a Th1 memory response specific to the translocated species [86]. In the scenario of bacterial translocation, live bacteria gaining access to spleen, lymph nodes, or tumor may initiate a strong immune response by providing both foreign antigens and adjuvants (MAMPs/PAMPs). Consequently, tumor cell killing may ensue due to T cell cross-reactivity or bystander activation within the tumor microenvironment (TME). Thus, commensals might bolster anti-tumor immunity through both augmented *antigenicity* as well as *adjuvanticity*, as described below.

#### Augmented antigenicity due to cross-reactivity to bacteria and tumor antigens

Some data suggest a mechanistic role for T cell epitopes shared between bacteria and tumor cells [18, 89, 95]. Under this proposed model, cross-reactive T cells primed against bacterial antigens might exert anti-tumor effects either by providing help (CD4^+^ T cells) or through direct killing (CD8^+^ T cells). In a preclinical study, adoptive transfer of *B. fragilis-*reactive CD4^+^ T cells conferred enhanced tumor control and restored anti-CTLA-4 efficacy in GF mice [18]. Peripheral immune cells isolated from patients receiving immune checkpoint blockade (ICB) treatment and assayed for in vitro T cell IFN-γ production following stimulation with certain bacteria showed an association with progression-free survival (PFS), whereas non-specific T cell activation with polyclonal activators demonstrated no relation to ICB response [89]. Balachandran et al. found intra-tumoral and circulating T cell clones with specificity to both neoantigens and predicted cross-reactivity with microbial epitopes [95].

#### Adjuvanticity of MAMPs/PAMPs

Microbiota-derived MAMPs or PAMPs can traverse the mucosal barrier and enter the circulation. For instance, serum from healthy individuals was demonstrated to contain stimuli capable of activating a range of TLR and NOD receptors [96]. In the cancer context, bacterial LPS aberrantly entering the circulation following total body irradiation augmented the activity of adoptive T cell therapy in mouse models [97]. Additionally, nucleic acids from bacteria have also been shown to act as natural adjuvants [98]. In particular, the unmethylated CpG dinucleotides enriched in prokaryotes are potent activators via TLR9. These pro-inflammatory microbial products can trigger at least partial activation of innate immune cells such as DCs. Such conditioned APCs might possess enhanced capacity to prime anti-tumor T cells. Evidence for heightened DC activation stemming from distinct microbiome compositions was illustrated in Sivan et al. who showed that splenic DCs isolated from mice colonized with *Bifidobacterium* sp. showed superior priming of naïve CD8^+^ T cells ex vivo [17]*.* Enrichment in *Faecalibacterium* genus in patients with metastatic melanoma associated with responsiveness to ICB therapy was also associated with increase in antigen processing and presentation markers in the tumor [90].

### Microbial metabolites as messengers

Gut bacteria produce various bioactive molecules as byproducts of their metabolism. These metabolites can exhibit diverse effects on the host, including modulating the immune system [99]. SCFAs are one of the most extensively characterized classes of microbial metabolites known to shape host immunity [100]. Through anaerobic fermentation, bacteria break down complex carbohydrates into SCFAs such as acetate, butyrate, and propionate. These metabolites are the primary energy source consumed by intestinal epithelial cells [101] and can also affect cytokine production [102], macrophage and DC function [59, 103], and B cell class switching [104]. SCFAs can additionally act to inhibit histone deacetylases, facilitating Treg differentiation [105]. By mimicking human signaling molecules, SCFAs can also act as ligands for G-protein coupled receptors [106]. Other bacterial metabolites relevant to host immunity include retinoic acid and co-metabolites, such as polyamines and aryl hydrocarbon receptor ligands [107]. These small molecules can impact immunity by acting as signaling molecules, epigenetic regulators, and metabolic switches and may ultimately shape anti-tumor immunity.

Given the predicted importance of bacterial metabolic contribution to host immunity and immunotherapy efficacy, there is significant interest in identifying both the specific bacteria exerting immune modulatory effects, as well as the functional and metabolic characteristics of these bacteria*.* To address this question, metagenomic and metatranscriptomic sequencing approaches coupled with metabolomic analysis of patient serum and stool samples will be critical for a more complete characterization of the biosynthetic pathways present within a given microbiome. Insights into metabolic contributions of the microbiome in the context of immunotherapy also may lead to new candidate therapeutic strategies, either through provision of desired metabolites as drugs, or via genetic manipulation of selected commensals for clinical administration.

### Host cytokines as messengers

Another potential mechanism by which gut bacteria could modulate systemic immune responses is through local induction of soluble immunomodulatory factors that then disseminate systemically. Circulating cytokines may shift the activation threshold of key immune subsets within the TME or TdLN, thus leading to augmented adaptive immune responses in the context of immunotherapy. Candidate mechanisms include increased production of type I interferons, IL-12 and TNFα, or decreased production of immune suppressive cytokines such as IL-10 and TGF-β. As an example, segmented filamentous bacteria can induce secretion of IL-22 from type 3 innate lymphoid cells in mice, causing production of serum amyloid A in the terminal ileum which, in turn, acts on the LP DCs to drive Th17 polarization [63, 108]. In cancer models, oral administration of *Akkermansia muciniphila* improved the efficacy of PD-1 blockade in an IL-12–dependent manner in mice [89].

### Immune cells as messengers

A recurring theme in many of the described mechanistic studies is that innate immune cells, often DCs, represent the central cell type affected by perturbations within the commensal community [17, 18, 85, 86, 109, 110]. DCs are key microbial sensors that bridge innate to adaptive immunity and are also critical for molding T cell responses within the TME. Microbial signals might only need to function locally in the LP and MLN to drive DC function and the subsequent delivery of the immunomodulatory effect to the TME might be carried out by the DCs themselves or downstream by T cells. Various innate immune cells have been shown capable of exiting the intestinal LP and translocating to the spleen and peripheral lymph nodes under steady state [111].

Different mechanisms of microbial sensing by DCs might be in play in the context of a damaged versus intact intestinal barrier. Compromised barrier integrity could allow for translocation of live bacteria or microbial products into the circulation. These could then be recognized by PRRs on innate immune cells, such as DCs, and affect downstream innate and adaptive immunity. Such potential mechanisms may contribute to microbiota-mediated modulation of anti-tumor immunity in situations of gut inflammation, such as with total body irradiation, chemotherapy agents that cause mucositis, or with anti-CTLA-4 treatment where 11% of patients experience colitis and 34% develop diarrhea [112]. However, anti-PD-1 therapy shows only 2% incidence of colitis [112], suggesting that additional mechanisms likely exist, by which commensals shape host immunity. On the other hand, in the context of an intact barrier, mucosal DCs constantly sample bacterial-derived antigens via various mechanisms. For instance, a subset of DCs in the LP are reported to be capable of extending dendrites between epithelial cells to sample the lumen [113]. DCs may also acquire proteins via goblet cell channels [114] or microfold cells (M cells) [115]. Bacterial antigen-loaded DCs could induce immune tolerance to commensal bacteria, or they could prime bacterial antigen-reactive T cells, which in some instances might be capable of cross-reacting with tumor antigens [18, 89, 95] or in other cases might provide bystander help during anti-tumor responses. In this respect, understanding the mechanisms driving tolerogenicity vs. immunogenicity might provide insight into the mechanisms of microbiota impact on antitumor immunity.

Given the complexity of the commensal-host interaction, the diversity of the microbiome, and inter-individual variability, it is likely that multiple modalities contribute to the impact of the microbiota on immunotherapy efficacy. Furthermore, the relative contribution of the microbiome will need to be integrated along with other dimensions affecting the potency of immunotherapy, including germline genetic determinants and tumor cell-intrinsic oncogenic alterations [116–118]. Determining the relative contribution of all these factors and the most translatable aspects to human health will require careful experimental design in cancer patients to test hypotheses stemming from murine experiments.

## Potential future clinical applications

### Use of antibiotics in conjunction with immunotherapy

The collective evidence linking the gut microbiome to immunotherapy efficacy creates exciting opportunities to improve clinical treatment strategies. A straightforward implication is that antibiotics administration to patients receiving cancer immunotherapies should be pursued with caution. Routy et al. found that antibiotics administration to patients in conjunction with immunotherapy was associated with shorter PFS and shorter overall survival (OS) [89] and these results have recently been supported by an additional retrospective analysis [119]. Additionally, greater bacterial diversity was associated with higher response rates to anti-PD-1 therapy [89, 90]. These data among others (reviewed in [120]) suggest that antibiotics may have detrimental effects on patient outcomes with checkpoint blockade immunotherapy, which should prompt discretion in their administration. However, one could also imagine that some patients may have an abundance of bacterial entities that dominantly promote immune suppression, such as through expansion of FoxP3^+^ Tregs. In those defined instances, appropriate antibiotics might decrease the abundance of such immune regulatory bacteria, perhaps allowing immune-potentiating bacteria to bloom and support improved tumor control. Studies are ongoing in reconstituted GFM to test these ideas.

### Use of the microbiome as a prognostic biomarker

The modulatory effects of the microbiome could foreseeably offer multiple avenues of clinical intervention. Microbiome composition could be considered as a complementary prognostic or predictive biomarker for treatment outcomes. Higher bacterial diversity in the gut (but not the oral microbiome) was identified to be associated with better response rates to ICB [90]. More specifically, certain bacteria were found to be enriched in anti-PD-1 responders while other species were enriched in non-responders. These data suggest that fecal DNA sequencing prior to therapy, by quantifying the community richness and the relative proportion of putatively identified “beneficial” or “detrimental” bacteria, may be suggestive of outcome and ultimately help guide treatment decisions. Prospectively designed clinical studies to validate these associations will be key to define the utility of these approaches. In the future, the composition of the microbiome may be one parameter incorporated with other known correlates of outcome such as T cell infiltration and tumor mutational burden to 1) predict potential efficacy with a given immunotherapy and 2) inform additional interventions via the microbiota to improve immunotherapy potency or alternatively decrease treatment related toxicity.

### Therapeutic interventions to modulate microbiome composition and function

**Fig. 2:**
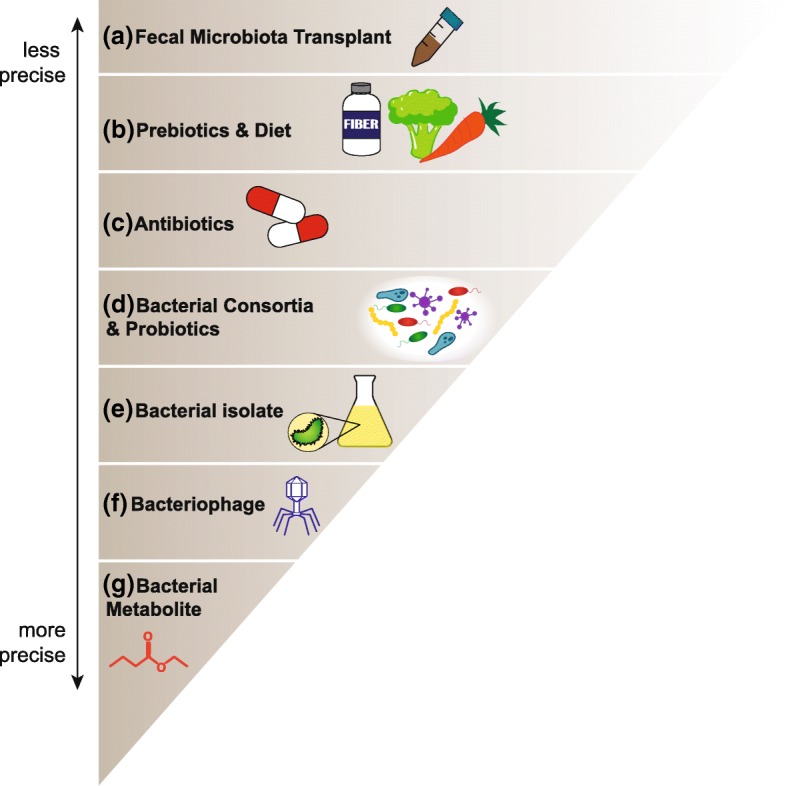
Microbiota-oriented interventions to improve immunotherapy treatment. While stable on a global scale, the gut microbiota regularly undergoes small fluctuations and is amenable to strategies which could shape the commensal community to either help improve patient response rates to immunotherapy or prevent treatment-related toxicity such as colitis. These approaches range from complex community transfers in the form of (**a**) fecal microbiota transplantation (FMT) which may have many effects on the recipient, to delivery of (**g**) a single microbial metabolite with a specific immune-modulatory effect. Additional approaches include (**b**) modulating macronutrient or prebiotic intake to shift bacterial communities, (**c**) targeting broad classes of bacteria with antibiotics, (**d**) administration of a select number of known beneficial bacterial species, or (**e**) a single defined bacterial isolate. Bacteriophages (**f**) or viruses that infect and kill selected bacteria, could also be used as a means of selectively depleting a detrimental bacterial population

Preclinical evidence extends the correlative relationship between the microbiome and response observed in patients to support a causal role. This scenario opens the exciting possibility to improve efficacy by manipulating the gut flora. Intervention strategies range from less precise or “blunt” approaches to more targeted therapeutic approaches (described in Fig. 2).

One such approach is fecal microbiota transplantation (FMT). For example, fecal samples could be prepared from anti-PD-1 responders that show a favorable composition of commensal bacteria, then transplanted endoscopically or prepared for oral delivery into patients who are anti-PD-1-resistant and show an unfavorable composition of gut microbes. This approach would parallel the strategies being used to treat refractory *Clostridium difficile* infection in patients [121]. This approach delivers a complex community and the promise to transfer its beneficial effect. However, FMT is clouded by uncertainties related to the imprecise definition of a favorable microbiota, the possibility of delivering immune-regulatory bacteria, and the potential to transfer disease-promoting bacteria such as those contributing to obesity or even carcinogenesis.

A subtler means of intervention may include modulating the existing commensal community via prebiotics or dietary changes to favor the expansion of beneficial bacteria that require specific substrates, or conversely, “starving” detrimental bacteria of their required nutrients. For example, short-term changes in human macronutrient consumption towards a high fat, low fiber animal-based diet increased bile-tolerant microorganisms (*Alistipes*, *Bilophila* and Bacteroides) and decreased levels of Firmicutes that metabolize dietary plant polysaccharides (*Roseburia*, *Eubacterium* *rectale* and *Ruminococcus bromii*) [122]. Similarly, antibiotics could be considered a means of targeting immune-regulatory bacteria. Both of these approaches lack the precision to modulate very specific bacterial populations, however, and may have variable effects depending on the starting state of the commensal community.

Alternatively, beneficial or immune-potentiating bacteria could be prepared as a probiotic and provided as an immunotherapy adjuvant. Once molecular mechanisms are determined, genetic manipulation of the selected bacteria could be utilized to maximize beneficial effects. Historically, certain bacterial species have been some of the most amenable organisms to genetic manipulation, and the breadth of tools available to study and modify bacteria continues to expand. This technology allows the modification of a bacterium’s existing function or the introduction of completely novel genes [123]. For example, a *Bacteroides* strain modified to carry a gene cluster to utilize porphyran stabilized its engraftment into mice fed a porphyran-supplemented diet [124]. This strategy effectively creates a unique metabolic niche for the exogenous microbe and presents a potential means to facilitate probiotic efficacy. Bacteria may also be genetically modified to drive expression of a metabolite of interest [125]. For well-characterized bacteria such as *Escherichia coli*, genetic manipulation is routine, but for many human commensals, incomplete genomic information leaves fewer tools available for these strategies currently. To circumvent this limitation, it is possible to express bacterial genes of interest heterologously in common laboratory hosts such as *E. coli* or *Bacillus subtilis* [125]. An alternative approach to adding beneficial bacteria to the microbiota is selective depletion of harmful species from the community. Bacteriophages are viruses that can infect and kill bacteria and are naturally present in the microbiome where they play a key role in preserving community equilibrium. Some phages have been used preclinically to decrease pathogenic bacteria while leaving the commensal community intact, and could be further engineered to target certain bacterial species or strains [123].

Finally, if a bacterial metabolic pathway is identified along with defined metabolic products that mediate improved anti-tumor immunity and immunotherapy, then small molecule entities could be tested as candidate immune-potentiating drugs. In all cases, appropriately controlled clinical trials will be required to validate any potential microbiome-based therapy and to assess benefits and risks. Clinical trials to evaluate the impact of fecal microbiome transplant and probiotic administration with checkpoint inhibitors are already underway [126].

## Conclusion – the future for the microbiome and immunotherapy

Given the intricacy of the microbiome, it will be challenging to tease out the essential mechanistic elements in such a complex system. Even if two individuals harbor the same species of bacteria, there can be variation of each bacterium at the strain level, which could yield divergent functions upon interaction with the host. Moreover, two identical strains in two disparate communities may contribute differently to their collective consortium and thus function differently with respect to the host. As such, tremendous care will need to be taken when assigning specific functional attributes to given commensal bacteria. Furthermore, a large majority of focus on cancer immunotherapy and the microbiome has investigated the contribution of bacteria but has yet to thoroughly investigate non-bacterial components including viruses, fungi and protozoa. Evidence in non-cancer disease models has indicated that the mycobiome (fungi) and the virome (viruses) can regulate systemic immunity. For example, manipulation of the mycobiome by oral antifungal drugs increased the severity of allergic airway disease in mice and was dependent on gut-resident CX3CR1^+^ mononuclear phagocytes [127, 128]. The virome, encompassing bacteriophages, mammalian viruses, and the endogenous retroviruses, is estimated to contain ten-fold more particles than bacterial microbes [129]. Supporting the link between the intestinal virome and host immunity, alterations in viral communities have been observed in the context of human immunodeficiency virus [130], and inflammatory bowel disease [131] and have been associated with autoimmune disorders including Type 1 diabetes [132, 133]. Incorporating a pan-kingdom view of the microbiome will likely lead to a more holistic understanding its impact on cancer treatment.

Looking forward, it is important to recognize that the microbiome contributes only one dimension to the many facets that govern the interface between cancer and the host immune response. Cancer cells grow and evolve under the selective pressure of therapy, and molecular evolution of the tumor could still occur when the microbiome is manipulated to maximize immunotherapy efficacy. In addition, it is conceivable that the composition of the microbiome similarly may evolve over the course of cancer progression and therapy administration. This variation offers additional research challenges, but with this pliability also comes exciting promise for intervention and exploiting the host-microbiome interdependency to deliver more potent therapy. In the future, it will be important to consider the microbiota as one of several parameters to be incorporated into considerations of personalized cancer therapy.

## Abbreviations

Allo-HSCT: Allogeneic hematopoietic stem cell transplantation
APCs: Antigen-presenting cells
CTL: Cytotoxic T lymphocyte
CTLA-4: Cytotoxic T-lymphocyte-associated protein 4
DC: Dendritic cell
GF: Germ-free
GVHD: Graft-versus-host disease
ICB: Immune checkpoint blockade
LP: Lamina propria
MALDI-TOF: Matrix-assisted laser desorption ionization time-of-flight
MAMP: Microbe-associated molecular pattern
MDSC: Myeloid-derived suppressor cell
MLN: Mesenteric lymph nodes
OS: Overall survival
OTU: Operational taxonomic unit
PD-1: Programmed cell death protein 1
PD-L1: Programmed death-ligand 1
PAMP: Pathogen-associated molecular pattern
PFS: Progression-free survival
PRR: Pattern recognition receptor
PSA: Polysaccharide A
SCFA: Short-chain fatty acids
SFB: Segmented filamentous bacteria
SPF: Specific pathogen-free
TdLN: Tumor-draining lymph node
TME: Tumor microenvironment
